# A review on considerations needed educating new physicians

**Published:** 2015

**Authors:** A Alavi, N Amjadi

**Affiliations:** * Fertility and Infertility Research Center, Hormozgan University of Medical Sciences, Bandar Abbas, Iran,; ** Preventative Gynecology Research Center (PGRC), Shahid Beheshti University of Medical Sciences, Tehran, Iran

**Keywords:** medical education, public health, educational alternatives, physicians

## Abstract

The long process of medical education is important crucial parts related to public health. This research was led to study the educational alternatives in teaching new physicians.

The necessity for a revolution in the medical education is due to the increased number of medical students, constant number of constant number of cases, and their prospects enhanced professors workload and enhanced supervisory agency mistake less clinical events happening for medical students, interns and residents. Newer techniques have become available in improving education. Stimulation has been utilized before as an educational technique in military training, space programs, aviation industry, sports stimulation, nuclear power industry, teaching anatomy, anesthesiology, and resuscitation training. Physical models (in a skills lab or workshop), computer programs including (over the internet), standard patients, etc., can be utilized for this technique. By reviewing the literature, we can conclude that several subjects are needed to be explained earlier education. Thus, to conduct an accurate program, these questions must be answered, appropriately.

## Introduction

Medical education is a lengthy system that takes four to 12 years of education. Its a collection of lectures, tutorials, clinical experiences and night shifts and contains socialization experience, acquiring knowledge, attitudes, skills, values and sense of ethics [**1**-**3**].

The scope, type, and timing of medical schools vary worldwide. Recently, health services of many countries have felt the need to change their educational system to better prepare future doctors for their role and provide the highest quality of care to patients and modify current methods which are known to be dehumanizing, saddening, offensive and severe [**1**,**4**-**9**]. 

Due to the increased number of medical students, the constant number of cases and their prospects, and enhanced supervisory agency mistake, less clinical events are occurring for medical students, interns and residents [**1**,**10**]. Also, the increased professor’s workload reduces the opportunity to improve education. Thus, the current model of “learning by doing” has to change, especially because it involves real patients [**1**,**11**]. 

Education focused on specific skills or procedures can increase self-confidence and competence of the students [**11**]. Also, studies have explained that specific skills such as an a suitable connection among patients and their physician increases health outcomes (such as patient understanding and recall, symptom resolution, treatment adherence, and patient satisfaction) and decreases malpractice and medical errors [**4**,**12**-**14**] 1B. Also, fewer malpractice claims occur after a good communication [**15**-**17**]. These skills are important parts of practice and are carefully evaluated before granting practice license in many countries. Relying on few experiences that may or may not occur during rotations does not seem to be efficient and may lead to the unpreparedness of the future physicians [**18**]. 

There are many tasks required to master a procedure; developing cognitive abilities to understand evidence and contraindications, expanding technical expertise, learning to interpret the results, developing communicational skills and bedside manners, and how to obtain an informed consent [**19**]. Studies have shown that students have high anxiety and low levels of confidence performing various skills [**11**,**20**,**21**].

These core skills can be taught to students. However, there are many challenges that are needed to be defeated. The most important problem is the process of teaching a large number of students in a limited period. Due to the crowded curriculum of medical schools, several medical skills have not received their requiring dedicated timing and the relying on current resources has not efficiently solved this problem. Time shortages on both ends (students and educators) are a challenging part of the medical skill education [**11**,**20**]. Even among these skills, the personality development (including communicational skills, development of ethics sense, etc.) has been undermined. Performing these skills and mastering them during clinical experiences and patients’ exposures are the most often implemented teaching methods. However, due to variable and sometimes, lack of supervision [**19**,**22**]. 

A study conducted by Wickstorm et al. showed that residents had performed only 15.4 percent of the required procedures frequently enough to be confident during the procedures [**23**]. Wigton et al. also showed that less than half of the residency program directors believed that their residences had mastered basic procedural skills [**24**]. Other studies have shown that new residents should learn the procedural skills exactly as medical students. However, many institutions use their residents to teach these skills to medical students [**19**].

The time of the student teaching has never been this brief. However, studies have shown that a short course, even a one day course, can significantly improve the learners skills leading to a better health outcome and a significant increase in the physicians’ job satisfaction [**11**,**19**-**21**,**25**].

Previous studies have reported a reduced clinical experience for medical students and fewer case encounters [**1**], which led to a lower proficiency on implementing the required skills [**26**-**28**]. Another reason for fewer procedural experiences is that nurses, IV teams, phlebotomists and other hospital staff, routinely conduct basic skills [**6**,**19**]. Other than fewer clinical experiments for students, this leads to fewer opportunities for faculty members and many of them perform each procedure fewer than ten times per year. Thus, they feel uncomfortable to teach them to the collegians [**29**]. In turn, it results in fewer educational positions for the medical students since residents might lack the confidence or rather keep the experience for themselves. The unpreparedness of students creates two other obstacles; it is unethical to get the procedures on real and actual patients, and that patients are not willing to let trainees perform procedures on them. Another obstacle can be that students might not be comfortable to perform these tasks on patients [**28**,**30**,**31**]. 

Studies have shown that logging the amount of procedures can improve proficiency and provide further opportunities for students to perform procedures [**32**]. Students should be assessed to have a level of competence before performing procedures on patients. This assessment can be conducted by evaluating their ability to interpret the results of similar practice, ask the indications and contraindications to assess their knowledge, and finally, conduct the experiment on animal models or stimulated patients [**25**,**33**]. 

**Stimulation as an educational technique**

Stimulation has been used to teach various skills before. The first place that used stimulation for education was the military. Also, space programs, aviation industry, sports stimulation (such as chess, etc.), and nuclear power industry are other samples of stimulation implementation. In medical education, teaching anatomy, anesthesiology and resuscitation training have used stimulation for some time and theirs experience has shown to be prosperous [**34**-**36**]. 

Simulation can be conducted by using physical models (in a skills lab or workshop), computer programs inclusion (over the internet), standard patients, etc. This method offers many benefits. It provides a planned exposure to clinical situations and realistic learning environments. Students can repeat their experiences many times in a safe and controllable environment [**11**,**18**]. The utility of this technique has previously been proven in military training and for curing psychological disorders [**36**-**39**]. Learners can practice infrequent clinical conditions in a protected environment [**40**,**41**].

However, the limitation of information regarding this method might prevent many organizations from implementing this technique.

Stevens et al. conducted a study to investigate the usage of virtual patients in teaching clinical and communicational skills to medical students. They reported many problems in understanding the students’ queries which can lead to the student becoming exhausted and can result in education failure [**18**]. They concluded that using virtual patients can decrease the expenses compared to standardized patients and an endless repository of clinical scenarios can be made. They added that this method could comply with different educational needs and provide a safe educational environment for students. Participants of that study recognized the implementation of virtual instructor as a powerful educational tool [**18**].

Meyers et al. conducted a study to investigate the effects of teaching technical skills to medical students during their surgery clerkship. They assigned third year students to a three-week program and taught knot tying, foley catheter and nasogastrial tube placement, suturing, I.V. placement and arterial puncture. They also used a self-reported checklist to report their skill before and after the intervention. They showed that all skills were better performed after the intervention, however, the increase in NG tube insertion and removal were not significant. They concluded that introducing specific technical skills during clerkship could improve the skills of the students [**11**].

Stewart et al. conducted a study to determine the outcomes of pre clinical skills course on the proficiency, anxiety, and confidence of medical students while performing these tasks. The skills included reading electrocardiograph, abdominal and chest x-rays, obtaining bedside chart data and interpretation of them, knot tying, nasogastrial tube insertion and removing, suturing, sterile technique, obtaining an informed consent, intravenous catheterization and foley catheter placement and removal. A self-reporting checklist was distributed between the members, back and next arbitration. They showed that proficiency and self confidence of students significantly increased in all taught areas after the four hour course [**20**].

**Use of workshops to teach procedural skills**

Many medical schools have developed workshops that provide experiments [**21**]. These workshops are occasionally carried out for pre clinical students who are at their third or fourth year of medical school [**42**,**43**]. These centers can provide opportunities in an organized way and the educators are not significantly doctors [**44**]. However, non-physician trainers are carefully assessed and their competency is certified. Before conducting the procedure on patients, students must pass a relative quiz and perform the experiments on plastic models. After passing these steps, the student must execute the procedure on actual patients while being supervised and after successful attempts, students get authorized to perform those tasks [**19**].

**Use of standardized patients**

Some universities and medical education organizations use standardized patients to teach their students and increase their clinical experience. This method has its own advantages compared to real patients. However, the use of this method is limited due to its high expense [**18**]. 

**Use of computer and internet to teach the required skills**

Due to the rapid advancement of computer technology, computer and internet technology are considered for entering the main stream of health care education. After the introduction and development of internet, it has been considered as a powerful source of information for many fields, including physicians [**11**,**45**]. Providing an interactive and multimedia experience that stimulates a real patient, lets the learner act as the physician and submit his experience, can be useful to students, residents and even graduated and licensed doctors [**46**]. However, the use of internet to teach skills is limited and is mostly used for CME learning and the efficacy of teaching medical students and inexperienced learners should be examined [**47**,**48**]. 

A difficulty in utilizing this technology is that they eliminate the non-verbal skills and the student might fail to recognize it as a crucial part of the communication [**49**]. 

We reviewed few additional techniques that can help students and residents achieve competence in required skills. However, it must be kept in mind that none of them is enough and they can only replace very early stages of clinical education. Further studies are needed to evaluate the effects of teaching aids on patient care and health care costs [**44**].

## Discussion

Based on the reviewed literature, a number of questions should be answered prior to clinical education:

1. Which skills and procedures should be mastered by medical students or residents?

2. When should they start to learn the procedures and skills?

3. Who should teach the procedures and required skills to medical students? 

4. Which method should be used to teach these skills?

5. How many efficiency are required for competency? 

6. What items should be evaluated to see whether they have mastered the skills or not?

7. How should this assessment be done?

The type of skills that are necessary to learn should be reassessed in different environments [**50**]. 

Even though anybody who can teach these skills should be welcomed (such as fellow medical students, interns, residents, professors, etc.), it is best to train the students using hospital personnel who are actually involved with those specific procedures in a daily base; nasogastric tube and urethral catheter insertion and removal can be taught by nurses, placing intravenous catheters can be educated by IV teams, arterial punctures can be taught by respiratory therapists, laboratory staff can teach preparing specimens, and residents and attending can teach more specialized procedures [**4**,**11**,**19**,**51**,**52**]. However, as mentioned earlier, hospital staff involved in the medical training must be carefully assessed and certified.

McLeod et al. have suggested standards for teaching educational skills [**10**,**51**]: 1. Assessing the needs and preparing the student, 2. Clearly performing the techniques and providing educational commentaries, 3. Asking the student to perform the procedure and carefully observing the learner and encouraging repetition, 4. Providing appropriate feedback, 5. Pointing out the strengths and weaknesses while encouraging the students, 6. Developing various situations and scenarios for the students to repeat the practice, 7. Understanding that every learner’s skills and personalities are different and are prepared to modify the approach. 

Using a suitable evaluation system is important to assess student skills. Some studies have suggested self-assessment as a tool for the assessment of the proficiency of students and it is recognized as an essential part of skill building. Especially since students can review and analyze their own skills [**4**], However, various studies have shown that it is inefficient and students are inclined to exaggerate their capabilities [**52**-**56**]. Overall, since it allows faculty members to see what the students observe and value and thus, provide invaluable feedback, self assessment is an important tool and must not be forgotten [**4**].

Some studies suggest videotaped encounters with simulated or standard patients along with self-reporting tools to increase the precision and accuracy of the assessment [**57**-**62**].

The early introduction of additional methods to medical students can increase the chances of mastering the required skills. However, it is needed to carefully evaluate the concepts learned by these learning aids and assess whether they are applied on actual clinical experiences or not [**20**,**52**,**63**]. The final thing to remember is that despite the expansion of all these methods and techniques, none of them are able to replace the actual clinical experience and all these methods are only preparing students to achieve the maximum possible experience possible [**44**].

**Conflicts of interest**

The authors of this article notify that they have no conflicts of interest.

**Source of funding**

None
